# Unexpected evolutionarily conserved rapid effects of viral infection on oxytocin receptor and TGF-β/pSmad3

**DOI:** 10.1186/s13395-017-0125-y

**Published:** 2017-05-15

**Authors:** Yutong Liu, Irina Conboy

**Affiliations:** Department of Bioengineering and QB3 Institute, Univerisity of California, Berkeley, 174 Stanley Hall, Berkeley, CA 94720 USA

**Keywords:** Oxytocin Receptor, Viral Vectors, Viral infections, RNAi, Smad, TGF-β, ERK, Muscle Regeneration

## Abstract

**Background:**

shRNA lentiviral vectors are extensively used for gene knockdowns in mammalian cells, and non-target shRNAs typically are considered the proper experimental control for general changes caused by RNAi. However, the effects of non-target lentivirus controls on the modulation of cell signaling pathways remain largely unknown. In this study, we evaluated the effect of control lentiviral transduction on oxytocin receptor (OXTR) expression through the ERK/MAPK pathway in mouse and human skeletal muscle cells, on myogenic activity, and in vivo on mouse muscle regeneration. Furthermore, we mined published data for the influence of viral infections on OXTR levels in human populations and found that unrelated viral pathologies have a common consequence: diminished levels of OXTR.

**Methods:**

We examined the change in OXTR mRNA expression upon transduction with control and Smad3-targeting viral vectors through real time RT-PCR and Western blotting, and confirmed with immunofluorescence. Changes in Smad3 and OXTR expression were examined both in vitro with mouse and human myoblasts and in vivo in mouse satellite cells. The general effects of viral infections on OXTR downregulation in humans were also examined by analyzing published Gene Expression Omnibus (GEO) datasets. The change in myoblast myogenic activity caused by the viral transduction (the percent of Pax7 + Ki67+ cells) was examined by immunofluorescence.

**Results:**

Results shown in this work establish that lentiviral control vectors significantly downregulate OXTR expression at mRNA and protein levels and diminish key downstream effectors of OXTR, ERK signaling, reducing the myogenic proliferation of infected cells. This effect is evolutionarily conserved between mouse and human myogenic cells, and it manifests in satellite cells after control lentiviral transduction of mice in vivo. Furthermore, an examination of published datasets uncovered similar OXTR downregulation in humans that are afflicted with different viral infections. Additionally, cells transduced with Smad3-targeting shRNA downregulate OXTR even more than cells transduced with control viruses.

**Conclusions:**

Our work suggests that experimental cohorts transduced with control viruses may not behave the same as un-transduced cells and animals, specifically that control viral vectors significantly change the intensity of key cell-signaling pathways, such as OXTR/ERK. Our results further demonstrate that lentiviral transduction significantly decreases myogenic proliferation and suggest that viral infections in general may play a role in decreasing muscle health and regeneration, a decline in metabolic health, and a lower sense of well-being, as these rely on effective OXTR signaling. Additionally, our data suggest pathway crosstalk between TGF-β/pSmad3 and OXTR, implying that sustained attenuation of the TGF-β/pSmad3 pathway will reduce pro-regenerative OXTR/pERK signaling.

**Electronic supplementary material:**

The online version of this article (doi:10.1186/s13395-017-0125-y) contains supplementary material, which is available to authorized users.

## Background

RNA interference (RNAi) is a form of post-transcriptional gene silencing that is mediated by short double-strand RNAs known as small interfering RNA (siRNA). Experimental down-modulation of the canonical signal transduction by RNAi is a common practice used to access changes in cell behavior and hence, significance of a particular pathway. Control transduction viral particles typically are either empty vector backbones that do not activate the RISC pathway, or encode non-target, scrambled shRNA or shRNA to non-mammalian genes (GFP, for instance). The use of such negative control viruses presumes that there will be no cellular or organismal changes in key conserved cell-signaling pathways as compared to the viruses encoding shRNA that target specific pathway genes (e.g., Smad3, Notch, beta-catenin, etc.) [1]. Even when pathways are not themselves targeted, changes in their signaling intensity can impact the interpretation of the results. While lentiviral-mediated RNAi has proven to be efficient in gene silencing and knockdown, and control viruses serve well in this regard [1], little has been studied on how non-target lentiviral controls might affect the intensity of cell regulatory networks.

Previous studies have shown that canonical evolutionarily conserved signaling pathways, including Notch, TGF-β, MAPK, and Wnt, are involved in regulating embryonic organogenesis and adult tissue maintenance and repair [2–12]; skewing any of these pathways leads to a decline in tissue regenerative capacity [13–18]. For example, transforming growth factor beta (TGF-β) and the downstream pSmad3, have been shown to become elevated with age and inflammation, thereby suppressing muscle regeneration and adult neurogenesis [16, 19]. Oxytocin (OXT), on the other hand, a hormone that acts through the OXTR/pERK pathway, has been established as an indispensable determinant of muscle maintenance and regeneration that declines with age [20]. OXT signaling has been also implicated in bone maintenance, reduction of obesity [21–25] in addition to its better-known role in formation of trust and bonding, and enhancement of OXT signaling is used in clinical trials for combating autism and age-related dementia [26–30].

As part of a study whether there is a correlation between the TGF-β pathway signaling and oxytocin receptor (OXTR) expression, we used shRNA vectors targeting Smad3 as a means to downregulate the TGF-β pathway and examined the corresponding change in OXTR expression. We were interested to find that reducing Smad3 appears to negatively affect OXTR expression, indicating TGF-β signaling opposes OXT. Perhaps not surprisingly, control viral vectors did not reduce TGF-β receptors or Smad3. But, surprisingly, the controls for these studies: lentiviral vectors bearing a non-target, non-mammalian shRNA, or even just the viral backbone, significantly downregulated OXTR expression levels in primary myogenic precursors, which in itself would be predicted to reduce muscle maintenance and regeneration [20], and in vivo in muscle satellite cells.

This suggests that not only experimental cohorts but also the controls transduced with control viruses (in vitro and in vivo) are likely to differ from the un-transduced groups in the relative intensity of key regulatory signaling pathways. And it further implicates that viral infections may play a role in decreasing OXTR and thus broadly interfering with regeneration and maintenance of multiple tissues.

To follow this lead, a thorough, unbiased analysis of published GEO datasets revealed that humans afflicted with a number of different viral infections manifest a specific downregulation of OXTR expression, but not their cell surface receptors in general. In this regard, our data suggest that viral infections will decrease muscle and bone maintenance and repair, as well as the sense of well-being and psychological health, all of which rely on OXTR signaling.

Additionally, our study establishes that experimental attenuation of the TGFβ/pSmad3 pathway with Smad3-targeted shRNA, further downregulates OXTR, and correspondingly diminishes pERK, as compared to the non-target shRNA control vector. These data suggest a TGF-β/pSmad3 and OXTR pathway-pathway cross-talk and implies that a sustained inhibition of the TGF-β/pSmad3 pathway (that becomes elevated with age) will attenuate oxytocin function that is already diminished with age.

## Methods

### Animal strain

22–24-month-old C57BL/6 mice were obtained from NIH (Bethesda, MD, USA).

### Cell culture

Mouse primary myoblasts were cultured on Matrigel-coated dishes at 37 °C, 5% CO2 in growth medium (Ham’s F-10, Mediatech), penicillin/streptomycin antibiotics (500 IU/ml, 0.1 mg/ml; MP Biomedicals), and 20% Bovine Growth Serum (Life Technologies/Hyclone), supplemented with FGF-2 (6 ng/ml).

Human primary myoblasts were cultured on dilute Matrigel coated dishes (applied at 5 ug/cm^2 in phosphate-buffered saline (PBS)), at 37 °C, 5% CO2 in growth medium (Ham’s F-10; Mediatech), penicillin/streptomycin antibiotics (500 IU/ml, 0.1 mg/ml; MP Biomedicals), and 5% Bovine Growth Serum (Life Technologies/Hyclone), supplemented with FGF-2 (12 ng/ml).

### shRNA delivery by lentiviral transduction

In vivo transduction:

All procedures were performed in accordance with the administrative panel of the Office of Laboratory Animal Care, UC Berkeley. Old tibias anterior and gastrocnemius muscles were infected, in vivo, with non-target shRNA (Sigma SHC002V) control transduction lentiviral particles. Mouse Smad3 shRNA-producing lentiviral particles (from Sigma) were used for in vivo transduction experiments (target-set generated from accession number NM_016769.2):

1. Smad3-targeted shRNA1: CCGGCCCATGTTTCTGCATGGATTTCTCGAGAAATCCATGCAGAAACATGGGTTTTTG
2. Smad3-targeted shRNA2: CCGGCCTTACCACTATCAGAGAGTACTCGAGTACTCTCTGATAGTGGTAAGGTTTTTG
3. Smad3-targeted shRNA3: CCGGCTGTCCAATGTCAACCGGAATCTCGAGATTCCGGTTGACATTGGACAGTTTTTG.

Lot: 11191506MN; 7.6 × 10^6^ −1.4 × 10^7^ TU/ml stocks were used a MOI 0.3 for each administration of each shRNA-encoding virus. Lentiviral particles were delivered into skeletal muscle by intramuscular injections for two consecutive days, and muscle was injured with cardiotoxin on the second day. 3 days after the injury, the in vivo infected myofiber-associated satellite cells were isolated, cultured overnight, and studied by the RT-PCR.

In vitro mouse and human primary myoblast transduction

Primary mouse and human myoblasts were transduced in vitro for qRT-PCR, WB, and IF analysis. Primary myoblast cultures were transduced with different MOIs (see below) with Opti-MEM (Gibco by Life Technologies). Medium for control groups without any transduction was changed into Opti-MEM. 24 h later, medium containing lentiviral particles were removed from wells as well as the non-transducer control group, and fresh Opti-MEM were added to each group. Medium were changed every 24 h until sample collection.

Primary myoblasts were transduced with Smad3-targeted shRNA at MOI = 0.5, empty vector (SHC001V) at MOI = 0.005, 0.05, and 0.5, and with non-target shRNA (SHC002V) at MOI = 0.002, 0.02, 0.2, 0.5, 1, and 2. Viral titer for lentiviral particles are SHC001V (empty vector control): 7.7E7 TU/ml; SHC002V (non-target shRNA control): 2.6E7 TU/ml

### RNA extraction, RT-PCR, and real-time PCR

Total RNA was extracted from mouse and human primary myoblast cell culture using RNeasy Mini Kit (QIAGEN) according to manufacturer’s instructions. Total RNA was extracted from mouse gastrocnemius and tibialis anterior muscle using QIAshredder (QIAGEN) and RNeasy Mini Kit (QIAGEN) according to manufacturer’s instructions. Reverse transcription was performed with Invitrogen Superscript III First-Strand Synthesis System for RT-PCR according to manufacturer’s instructions. For real-time PCR amplification and quantification of gene of interest, 1 ug of total cDNA was used for the initial amplification using specific primers to each gene of interest; amplification was performed with a denaturation step at 95 °C for 10 min, followed by 40 cycles of denaturation at 95 °C for 15 s, and primer extension at 60 °C for 30 s. Real-time PCR was performed using Power*SYBR* Green PCR Mastermix (from Applied Biosystems) under QuantStudio3 (Applied Biosystems) and CFX Connect Real-Time System (Bio-Rad). Reactions were run in triplicates. Housekeeping gene GAPDH was used as an internal control to normalize the variability in expression levels, and results were analyzed using the 2^-ΔΔCT^ method described [31].

Primers used in real-time PCR:

- GAPDH1-F: GGGAAGCCCATCACCATCT
- GAPDH1-R: GCCTCACCCCATTTGATGTT
- GAPDH2-F: TGAGGCCGGTGCTGAGTATGTCGTG
- GAPDH2-R: TCCTTGGAGGCCATGTAGGCCAT
- Smad3 5′-F: CTGGCTACCTGAGTGAAGATGGAGA
- Smad3 5′-R: AAAGACCTCCCCTCCGATGTAGTAG
- Smad3 3′-F: ACACATTGGGAGAGGTGTGC
- Smad3 3′-R: GCAAGGGTCCATTCAGGTGT
- Smad3 shRNA-2-F: GCCTTACCACTATCAGAGAG
- Smad3 shRNA-2-R: AACCTTACCTCATCAGAGAG
- Smad3 shRNA-3-F: AACTCTCCAATGTCAACCG
- Smad3 shRNA-3-R: GCTGTCCAATGTCAACCG
- OXTR-F: GATGTCGCTCGACCGCTG
- OXTR-R: CGGTACAATGTAGACGGCGA
- TGFβR1-F: TCATTTCAGAGGGCACCACC
- TGFβR1-R: CAACTTCTTCTCCCCGCC
- TGFβR2-F: TGTATCTTGCCGTTCCCACC
- TGFβR2-R: CTCCACAGTGACCACACTCC

### Real-time PCR gel analysis

Four percent agarose gel was made using agarose from Fisher Scientific and TAE buffer from Biosciences. 6× loading dye from Fermentas was added to the final amplification product and 15 ul of the mixture was added to the gel. QuickLoad 100 bp DNA Ladder from New England Biolabs was used as reference. Pictures of the gel were taken with a BioRad GelDoc/ChemiDoc Imager and Quantity One software. Pixel density was then analyzed with ImageJ (NIH) by subtracting the background pixel density and normalizing each gene of interest to GAPDH respectively.

### Western blot analysis

Cells were lysed in RIPA buffer containing 1 mM PMSF, 1 mM sodium orthovanadate, PhosSTOP phosphatase inhibitor cocktail (Roche), and complete protease inhibitor cocktail (Roche). 30 ug of total protein extract in Laemmli buffer were resolved by SDS-PAGE on 10% precast gels (TGX, Bio-Rad) and were transferred to PVDF membranes (Millipore). Membranes were blocked for 1 h in 5% non-fat milk in PBST at room temperature. Primary antibodies against GAPDH (1:2000), actin (1:1000), pERK1/2 (1:1000), total ERK1/2 (1:1000), pSmad2/3 (1:1000), total Smad2/3 (1:1000), and OXTR (1:1000) were diluted in 5% non-fat milk in PBST. PVDF membranes were incubated in antibody solutions either overnight at 4 °C or 2 h at room temperature. Horseradish peroxidase-conjugated secondary antibodies were diluted 1:2000 in 1% BSA, and membranes were incubated for 1 h at room temperature. Blots were developed using ECL reagents (Advansta and Thermo Scientific) and were analyzed with Bio-Rad Gel Doc/Chemi Doc Imaging System and Quantity One software. Results of multiple assets were quantified by digitalizing the data and by normalizing pixel density of examined proteins by GAPDH pixel density with ImageJ (NIH).

### Antibodies

Primary antibodies:

- Smad2/3: Cell Signaling Technology #3102
- pSmad 2/3: Cell Signaling Technology #8828
- ERK1/2: abcam ab184699
- pERK1/2: Cell Signaling Technology #9101S
- GAPDH: abcam ab9485
- beta-actin: (ThermoFisher MA5-15739)
- OXTR: proteintech 23045-1-AP
- Secondary antibodies (all from Santa Cruz Biotechnology)
- Bovine anti-rabbit IgG-HRP: sc-2370
- Goat anti-rabbit IgG-HRP: sc-2004
- Donkey anti-goat IgG-HRP: sc-2020
- Bovine anti-goat IgG-HRP: sc-2350
- Bovine anti-mouse IgG-HRP: sc-2371
- Goat anti-mouse IgG-HRP: sc-2005
- Goat anti-rat IgG-HRP: sc-2006.

### Immunofluorescence

Myoblasts were cultured in chamber slides (LabTek CC2 coated glass) and were transduced with lentivirus as mentioned above. For OXTR staining, the slides were fixed in cold 4% paraformaldehyde (PFA) on ice for 5 min and were washed three times in PBS. For Ki67/Pax7 staining, the slides were fixed in 70% cold ethanol at 4 °C overnight. The slides were then permeabilized with 0.25% Triton-100 in PBS for 5 min, and were blocked in staining buffer (1% calf serum in PBS) for 2 h. Primary antibodies were added into staining buffer and were incubated for at least 4 h at room temperature or overnight at 4 °C. Three PBS washes were performed before secondary antibody were added and incubated overnight at 4 °C. Slides were then washed three times with PBS before mounted for fluorescence imaging.

Secondary antibodies:

Invitrogen Alexa Fluor 488 Donkey anti-Rabbit IgG (H + L) Secondary Antibody

Invitrogen Alexa Fluor 488 Goat anti-Mouse IgG (H + L) Secondary Antibody

### GEO dataset analysis

Patient studies for viral infections were identified from Gene Expression Omnibus datasets (GEO https://www.ncbi.nlm.nih.gov/geo/). Every dataset with information on OXTR, TGFβR1, or TGFβR2 were analyzed, regardless of cell types and types of viral infection. The fold changes of each gene measured in patients were normalized to non-infected control groups in a human study. For datasets with time-course experiments, time points starting and after 72 h were calculated. The accessions of GEO datasets used are as follows: GSE18816, GSE23031, GSE16593, GSE23031, GSE2067, GSE2067, GSE3292, GSE13597, GSE6802, GSE2815, GSE48466, GSE24533, GSE3397, GSE22589, GSE49954, GSE18816, GSE4785, GSE20755, GSE20948, GSE3397, GSE30719, and GSE27131.

### Data quantification

A non-paired, two-tailed *t* test was performed on all of the respective data. Quantified data were presented as means (SD). *P* values of < 0.05 were considered statistically significant.

## Results

### Control lentiviral vectors downregulates OXTR in mouse myogenic cells in vitro and mouse muscle stem cells in vivo

Lentiviral-mediated RNAi and transgenic gene expression have proven to be highly efficient and typically use empty vector backbones and non-target shRNA as controls. However, the influences of the vector controls on genes other than targeted were not well studied.

**Fig. 1:**
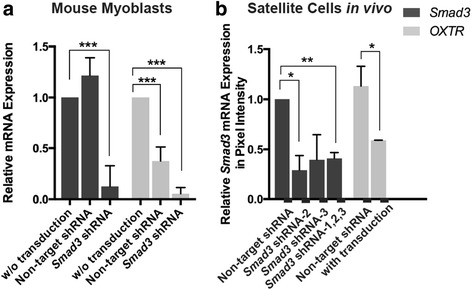
OXTR expression is downregulated upon lentiviral transduction. **a**. qRT-PCR in primary mouse myoblasts transduced in vitro with non-target (GFP) versus Smad3-targeting shRNA vectors at MOI = 0.5. OXTR expression is attenuated by all lentiviral vectors while Smad3 is attenuated only by Smad3-targeting shRNA vectors. **a**) Shows combined results from three shRNAs targeting Smad3. Results of individual shRNAs to Smad3 are shown in Figure 1SA. For mouse myoblasts: *N* = 6 for non-target shRNA, *N* = 8 for Smad3-targeting shRNA. **b**. OXTR is downregulated in muscle satellite cells in vivo with both non-target (GFP) shRNA and Smad3-targeting shRNA lentiviruses, but only Smad3 targeting diminished the Smad3 levels. Comparison between the level of OXTR in primary myoblasts and in vivo satellite cells is shown in Additional file 1: Figure S1B. *P ≤ 0.05, ** *P* ≤ 0.01, ***P ≤ 0.001, ****P ≤ 0.0001. For in vivo: * *P* ≤ 0.05; **P ≤ 0.01, ***P ≤ 0.001

We transduced primary mouse myoblasts with Smad3-targeting shRNA and non-mammalian, non-target shRNA controls and compared the expression levels of Smad3 and OXTR with those of primary mouse myoblasts receiving a mock transduction of no viral vectors. As expected, the non-mammalian shRNA control served as a reliable reference for the downregulation of Smad3 mRNA by the Smad3 targeting shRNA (Fig. 1a). However, interestingly, the non-target shRNA vectors significantly downregulated OXTR expression by ~70%, as compared to the un-transduced primary mouse myoblasts (Fig. 1a). Furthermore, the downregulation of Smad3 by the Smad3-targeted shRNAs further diminished OXTR expression, as compared to non-target shRNAs (Fig. 1a, Additional file 1: Figure S1A).

To examine if a similar phenomenon occurs in vivo, old C57.B6 mice, in which TGF-β/pSmad3 pathway becomes elevated with age, were injected intramuscularly with non-target non-mammalian (GFP) shRNA viral particles, versus three different Smad3-targeting shRNAs as in [30]. The transduced muscle satellite cells were isolated from the in vivo injected muscles 3 days later, as we have published [32]. The in vivo transduced satellite cells showed the expected downregulation of Smad3 when muscle was injected with Smad3-targeting shRNA viral particles, as compared to the non-target shRNA vector (Fig. 1b). Very interestingly, the in vitro observed trend of OXTR downregulation by viral transduction was also detected in these in vivo studies (Fig. 1b, Additional file 1: Figure S1B). Similar to the in vitro results, Smad3 shRNA in vivo additionally downregulated OXTR, as compared to the non-target shRNA viral vector (Fig. 1b).

While non-mammalian control shRNA vectors are designed to not target any known, canonical mammalian sequences, the presence of shRNA will engage with and activate the RNA-induced silencing complex (RISC). Interested in whether the downregulation of OXTR comes from viral transduction or RISC activation, primary mouse myoblasts were transduced with an empty vector (encoding just the viral backbone) that does not contain a hairpin insert, or with the above non-mammalian (GFP) shRNA control particles. OXTR expression levels were compared with those of non-transduced primary myoblasts. Our data shows that both control vectors caused a significant (~70%) downregulation in OXTR expression even at MOI = 0.05, which is much lower than the titer that is typically recommended for viral transductions; and that control shRNA particles downregulated OXTR faster than the viral backbone particles (Additional file 1: Figure S2A, B).

These data establish that transduction with control viral vectors perturbs key cell signaling molecules, such as OXTR at low viral titers.

### OXTR downregulation upon lentiviral transduction is evolutionarily conserved between mice and humans, and manifests upon different viral infections in human populations

**Fig. 2:**
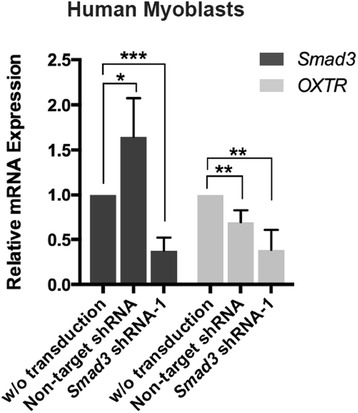
OXTR downregulation upon lentiviral transduction is evolutionarily conserved between mice and human. Shown are real-time qRT-PCR results on RNA derived from primary human myoblasts, which were transduced in vitro with non-target (GFP) versus Smad3-targeting shRNA vectors at MOI = 0.5. OXTR expression is attenuated by both lentiviral vectors while Smad3 is attenuated only by Smad3-targeting shRNA vectors. This downregulation of OXTR by lentiviral transduction is evolutionarily conserved between mouse and human

Interested in exploring the conservation of the observed phenomena between mouse and human cells, we repeated the above in vitro experiments with primary human myoblasts. Transduction of these human muscle precursors with non-mammalian, non-target (GFP) shRNA controls showed significant downregulation in OXTR expression, suggesting that OXTR downregulation upon lentiviral transduction is evolutionarily conserved between mouse and human. And similar to the outcomes with mouse myoblasts, this attenuation became stronger when a Smad3-targeting shRNA vector was used (e.g., when human Smad3 expression was experimentally diminished (Fig. 2B)). An examination of the complementarity demonstrated that mouse and human Smad3 loci have very high homology in the area targeted by this specific shRNA (Additional file 1: Figure S3) consistently with our data that this specific Smad3 shRNA targets Smad3 in both species.

Extrapolating these data, we asked whether OXTR downregulation would generally be caused by viral infections in humans and analyzed published GEO databases from human studies that focused on different viral infections (HIV, SIV, influenza virus, etc.). Specifically, we data-mined previously published GEO databases that report the levels of OXTR, Smad3, TGFBR1, and TGFBR2, which were analyzed with statistical rigor in the presence of various viral infections (in various cell types), as compared to the non-infected control groups within each human study.

**Fig. 3:**
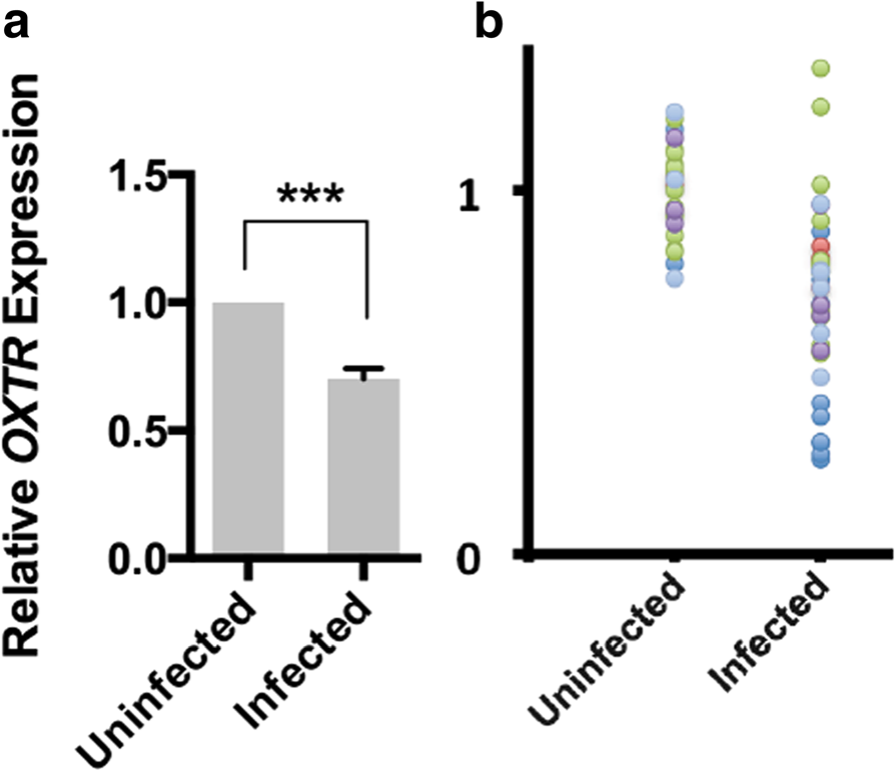
Human database analysis reveals downregulation of OXTR expression upon different viral transfections. **a.** Analysis of gene expression databases [34–48] shows statistically significant downregulation of OXTR in infection-afflicted humans, as compared to healthy individuals. ***P < 0.001. **b**. Scatter plots showing data from different databases designated by different colors. *Dots* are the OXTR levels in individual samples. Data obtained from every database showed diminished OXTR upon HIV, SIV, influenza virus, etc., infections, ranging from 47 to 82% downregulation, as compared with healthy control expression level. (OXTR: mean:0.475, SEM: 0.026; mean: 0.798, SEM: 0.019; mean: 0.826, SEM: 0.016; mean: 0.714, SEM: 0.043; mean:0.716, SEM: 0.018)

Interestingly, this analysis confirmed and extrapolated our conclusions by revealing that OXTR becomes significantly downregulated upon different unrelated viral infections in human populations (Fig. 3a). In addition to pooling the results from all databases, the data from each individual database were also examined: for each group, OXTR showed downregulation upon viral infections ranging from 47 to 82%, as compared with the level of this receptor in healthy individuals (Fig. 3b).

These results demonstrate an evolutionary conservation of OXTR attenuation by experimental and natural viral infections and indicate a significance of the uncovered phenomenon for human health.

### Lentiviral transductions do not downregulate cell surface receptors broadly

**Fig. 4:**
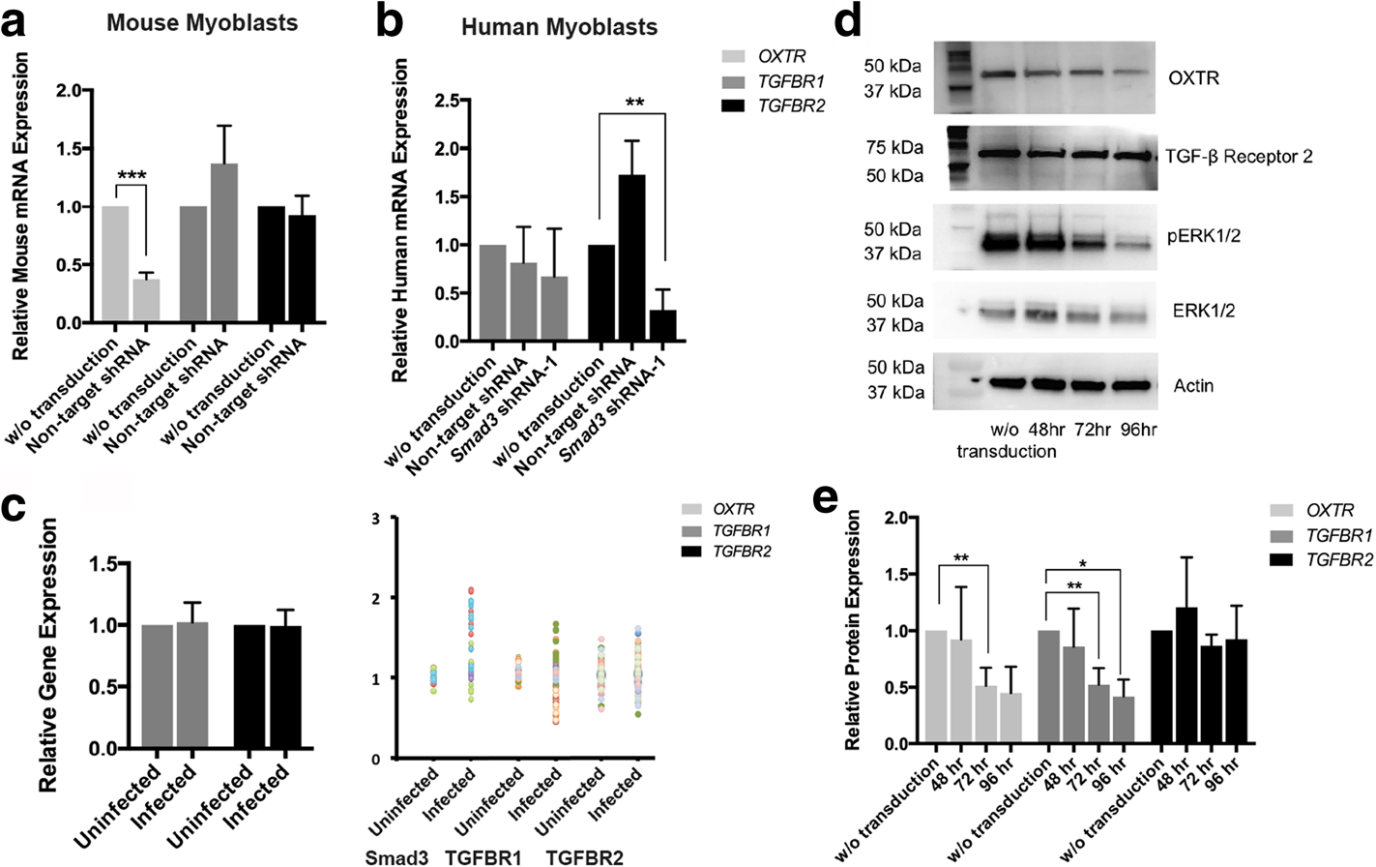
Viral transductions or infections do not universally downregulate cell surface receptors. **a**. qRT-PCR in primary mouse myoblasts transduced in vitro with non-target GFP shRNA vector at MOI = 0.5; TGFBR1 and R2 mRNA levels were not significantly changed upon transduction. **b**. qRT-PCR in primary human myoblasts transduced in vitro with non-target GFP shRNA and Smad3-targeting vectors at MOI = 0.5; unchanged TGFBR1 expression and elevated TGFBR2 expression were detected in three independent experiments with each cohort. **c**. Analysis of GEO human population gene expression databases [34–48] reveals no significant change of TGFBR1 and R2 in people upon viral infections. Scatter plots of different databases showed little variation in the expression in TGFBR1, but increased variation in TGFBR2 after viral infections and increase in Smad3 expression in some studies (while others showed no significant change in Smad3). (Smad3: mean:1.101, SEM:0.004; mean:1.702, SEM:0.011; Mean: 0.979, SEM: 0.012; Mean: 1.611, SEM: 0.115; mean: 1,042, SEM: 0.023); *P ≤ 0.05, **P ≤ 0.01, ***P ≤ 0.001. **d**. Western blotting analysis of OXTR, pERK1/2, and TGF-β receptor 2 in un-transduced primary mouse myoblasts and mouse myoblasts transduced with non-target shRNA lentiviral vectors. Compared with un-transduced cells, virally transduced myoblasts showed reduction OXTR and pERK1/2 protein levels that became more significant with increased time, while TGF-β receptor 2 levels did not change. Three individual western blots were quantified by pixel intensity. OXTR was normalized to actin, and pERK was normalized to ERK. (*P ≤ 0.05; ** *P* ≤ 0.01, ***P ≤ 0.001)

To answer whether the downregulation of receptors by non-target shRNA vectors applies to only certain proteins exemplified by OXTR, or manifests more generally, we examined the levels of TGF-β receptors 1 and 2 (TGFBR1 and TGFBR2) in primary mouse and human myoblasts by real-time qRT-PCR and western blotting. Interestingly, in contrast to the OXTR, expression levels of TGFBR1 and 2 were not significantly altered upon control lentivirial transduction in either mouse or human myogenic cells, and TGFBR2 actually became elevated in human myoblasts (Fig. 4a, b).

Moreover, while OXTR was significantly downregulated (Fig. 3), the expression levels of TGFBR1, TGFBR2, and Smad3 genes were not diminished in people afflicted with viral infections, as per our data mining study with the above-described human GEO databases (Fig. 4c). As for Smad3 expression, some human studies showed an obvious upregulation in expression upon viral infection, while others showed no change from healthy controls (Fig. 4c).

The expression levels of OXTR were further examined at protein levels through western blotting and were found to be reduced after the control lentiviral transduction, in a time-dependent manner (Fig. 4d). This downregulation of OXTR was further verified by immunofluorescence (Additional file 1: Figure S4). Furthermore, a key signaling molecule down-stream of OXTR, pERK [20], became attenuated concordantly with OXTR and with the same timing (Fig. 4d). In contrast, the protein levels of TGFBR2 did not change significantly, in agreement with our qRT-PCR results.

These results establish that viral infection promotes selected downregulation of certain, but not all cell surface receptors, which may perturb signaling networks. More specifically, these data suggest a skewing from OXTR/pERK to TGF-β/pSmad3 signaling upon viral infections, which is reminiscent of the age-imposed changes that associate with broad degenerative pathologies [17]. Interestingly, in human populations such conserved pathway skewing took place regardless of the specific viral infections or their distinct mechanisms of action or diseases.

### Viral infections decrease myogenic activity in vitro

Since OXTR signaling plays a major role in maintaining myogenic activity and muscle health [20], we hypothesized that control viral transductions, which attenuate OXTR, would also diminish the myogenicity of even young muscle progenitor cells.

**Fig. 5:**
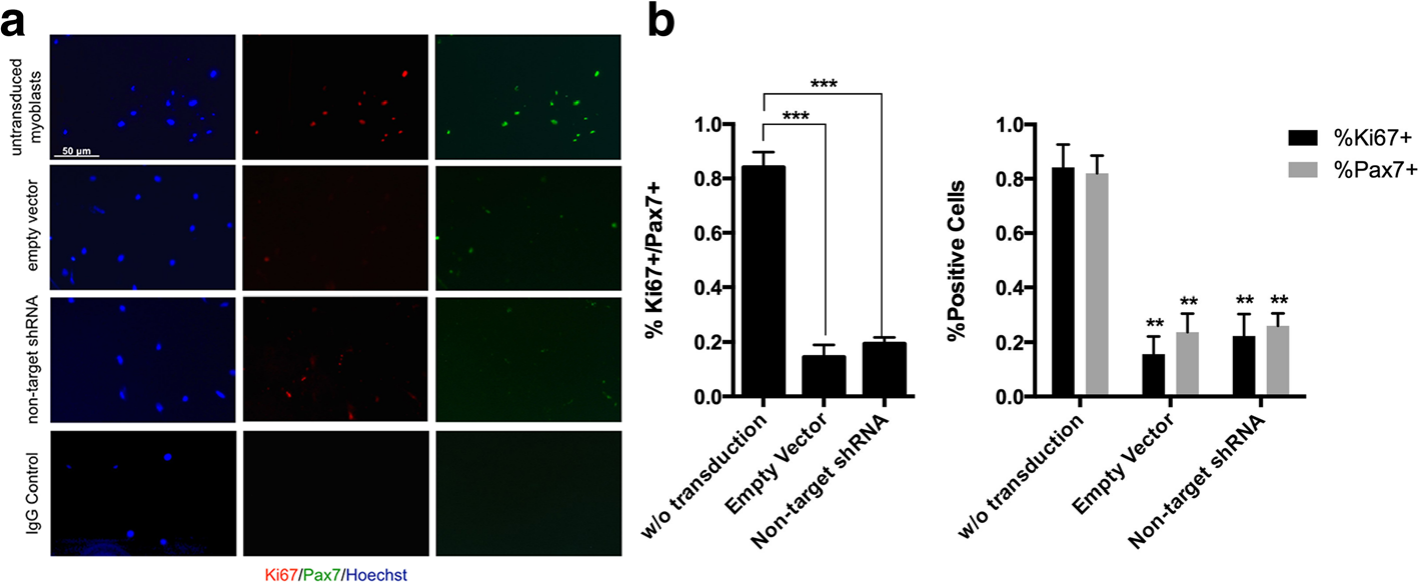
Lentiviral transduction decreases myogenic activity in primary mouse myoblasts. **a**. Representative 20× IF images for Hoechst (*blue*), Ki67 (*red*), and Pax7 (*green*). Percent of proliferative myogenic cells Ki67+/Pax7+ were quantified out of total Hoechst + cell numbers and were found to be significantly decreased after transduced with either empty vector particles or non-target GFP shRNA vectors. **b**. Number of single positive (Pax7+ or Ki67+) cells out of total Hoechst + cells were quantified and each population was diminished by either empty vector particles or non-target GFP shRNA vectors. (*N* = 3, ** *P* ≤ 0.01 ***P ≤ 0.001)

To examine this hypothesis, we compared the in vitro proliferation of myogenic (Pax7+) cells that were derived from young mice. Specifically, primary mouse myoblasts were transduced with either empty vector (encoding just the viral backbone) or with non-mammalian (GFP-encoding) shRNA particles and were compared with the mock-transduced cohort. All cells were fixed at 5 days after the transduction and were analyzed by co-immunodetection of Pax7+ and the proliferation marker Ki67 (Fig. 5a). The percentage of Ki67+/Pax7+ double positive, as well as Ki67+ and Pax7+ single positive myoblasts out of all Hoechst + cells were quantified and compared between the cohorts (Fig. 5b).

Interestingly and in complete agreement with our above observations, as compared to the un-transduced myoblasts, transduction with either the viral backbone or with the control shRNA particles dramatically and with high statistical significance reduced the percent of proliferating primary muscle progenitor cells from ~85% down to ~20%. Additionally, the numbers of cells expressing Pax7+ also significantly declined, proliferating or not (Fig. 5b), suggesting that viruses inhibit this key myogenic transcriptional factor [33]. When measured separately from the myogenic marker Pax7, the cell proliferation, e.g., percent of Ki67+ cells, has also declined upon the control viral transduction (Fig. 5b), which is consistent with the inhibition of p21 by OXTR [20]. Since so few cells were proliferating, it is unlikely that Pax7− cells overtook the culture during this experiment.

These data suggest that viral infections might play a negative role in skeletal muscle maintenance by suppressing myogenic proliferation and may lead to the loss of muscle stem cell identity through downregulation of Pax7 [33].

## Discussion

Numerous studies have been conducted with viral backbone and non-targeting shRNA vectors in mammalian cells and in vivo, and these have been assumed to control for changes in cell behavior and gene expression as compared to the experimental gene delivery. The data presented in our work shifts this paradigm by demonstrating that non-target non-mammalian shRNA encoding and even viral backbone-encoding viruses significantly downregulate a canonical cell-signaling pathway (OXTR-ERK) and perturb the TGF-β/OXT balance. Notably, our results show that cells and animals exposed to control viruses (in vitro and in vivo) will differ from un-transduced cohorts in a number of ways.

OXTR positively regulates muscle stem cell responses through the MAPK/ERK pathway, which among other broad effects attenuates CDK inhibitors and promote myogenic proliferation [20]; and in addition to its role in muscle health, oxytocin-OXTR is a determinant in trust and bonding, child bearing, mental well-being, and metabolic homeostasis, and it is also implicated in bone health and metabolism [21–26]. In this regard, our data suggest that in addition to known pathological manifestations, viral infection may play a less direct role in decreasing the health and regeneration of skeletal muscle, bone, brain-psychological well-being and inducing obesity by skewing key cell signaling networks that regulate homeostatic tissue maintenance.

While we show that viral infections and experimental transductions do not broadly attenuate all cell-surface receptors, it is likely that more than just OXTR becomes downregulated. For example, Pax7 rapidly declined in primary myoblasts transduced with the backbone or GFP shRNA control vectors. In this regard, it would be interesting to examine whether and how Pax7 and other myogenic cell-fate control genes [33] experience a virally induced attenuation (OXTR-dependent or by a parallel pathway).

Finally, our study establishes that TGF-β/pSmad3 and OXTR pathways actively interact and imply that sustained attenuation of the TGF-β/pSmad3 pathway (elevated with age) might be not entirely positive for tissue health and maintenance, as with time it will block the function of the hormone oxytocin.

## Conclusions

Lentiviral control vectors downregulate the OXTR/ERK signaling, allowing the TGF-β/pSmad pathway to become prevalent. This phenomenon is evolutionary conserved between mice and humans; and it manifests in people who suffer from different viral infections. Skewing from OXTR to TGF-β/pSmad is predicted to diminish the maintenance and repair of skeletal muscle (and possibly, other tissues), and accordingly, control viral infections diminish myogenicity.

## Additional file

Additional file 1: Figure S1. Additional comparative qRT PCR data. **a.** The in vitro studies with mouse primary myoblasts that were transduced with shRNA 1, 2, 3, and their mix. **b.** Comparative qRT PCR on un-transduced myoblasts vs. satellite sells, vs. control shRNA in vivo transduced satellite cells. *N* = 3, *,**, *** *P* < 0.05. **Figure S2.** Dose curve and timing of OXTR downregulation by control viruses. **a.** Non-target shRNA and empty vector particles downregulate OXTR in a dose-dependent manner. OXTR downregulation increases with viral dosage with both empty vector particles and non-target shRNA vectors. Myoblasts transduced with non-target shRNAs were collected at 72 h, and those transduced with empty vector particles at 120 h. OXTR downregulation is significant even at low viral dosage. *N* = 3 for each cohort. ****P* < 0.05. **b.** Empty vector particles take longer to decrease OXTR expression compared with non-target shRNAs, but in both cases significant downregulation is detected by 120 h post transduction. *N* = 3 for each cohort and each time point. ****P* < 0.05. **Figure S3.** High sequence homology between human and mouse Smad3 loci in the area targeted by shRNA are observed and are demonstrated by the alignment. **Figure S4.** Lentiviral transfection downregulates OXTR as determined by the immunofluorescence. Immunofluorescence for OXTR (*green*) was performed with un-transduced primary human myoblasts versus human myoblasts transduced with control lentiviral shRNA vectors. Hoechst (*blue*) labels all nuclei. OXTR-specific immunofluorescence was diminished in primary human myoblasts transfected with non-targeted shRNA vectors, as compared to the un-transduced cells. **Figure S5.** Gel confirmation of qRT PCR results. Amplification products from the real-time RT-PCR reactions were run on a gel, and the pixel intensities of each band were quantified by normalizing to *GAPDH*. **P* ≤ 0.05; ***P* ≤ 0.01. (PDF 4347 kb)

## Abbreviations

OXT: Oxytocin
OXTR: Oxytocin receptor
RNAi: RNA interference
siRNA: Small RNA
Smad3: Mothers against decapentaplegic homolog 3
TGFBR1: TGFβ receptor 1
TGFBR2: TGFβ receptor 2
TGF-β: Transforming growth factor beta
