# Supplementary material for: Unexpected evolutionarily conserved rapid effects of viral infection on oxytocin receptor and TGF-β/pSmad3
Source: Skelet Muscle. 2017 May 15;7:7. doi: 10.1186/s13395-017-0125-y (PMC5433165; doi:10.1186/s13395-017-0125-y)
Supplement: Additional file 1: Figure S1. — Additional comparative qRT PCR data. a. The in vitro studies with mouse primary myoblasts that were transduced with shRNA 1, 2, 3, and their mix. b. Comparative qRT PCR on un-transduced myoblasts vs. satellite sells, vs. control shRNA in vivo transduced satellite cells. N = 3, *,**, *** P < 0.05. Figure S2. Dose curve and timing of OXTR downregulation by control viruses. a. Non-target shRNA and empty vector particles downregulate OXTR in a dose-dependent manner. OXTR downregulation increases with viral dosage with both empty vector particles and non-target shRNA vectors. Myoblasts transduced with non-target shRNAs were collected at 72 h, and those transduced with empty vector particles at 120 h. OXTR downregulation is significant even at low viral dosage. N = 3 for each cohort. ***P < 0.05. b. Empty vector particles take longer to decrease OXTR expression compared with non-target shRNAs, but in both cases significant downregulation is detected by 120 h post transduction. N = 3 for each cohort and each time point. ***P < 0.05. Figure S3. High sequence homology between human and mouse Smad3 loci in the area targeted by shRNA are observed and are demonstrated by the alignment. Figure S4. Lentiviral transfection downregulates OXTR as determined by the immunofluorescence. Immunofluorescence for OXTR (green) was performed with un-transduced primary human myoblasts versus human myoblasts transduced with control lentiviral shRNA vectors. Hoechst (blue) labels all nuclei. OXTR-specific immunofluorescence was diminished in primary human myoblasts transfected with non-targeted shRNA vectors, as compared to the un-transduced cells. Figure S5. Gel confirmation of qRT PCR results. Amplification products from the real-time RT-PCR reactions were run on a gel, and the pixel intensities of each band were quantified by normalizing to GAPDH. *P ≤ 0.05; **P ≤ 0.01. (PDF 4347 kb) [file 13395_2017_125_MOESM1_ESM.pdf]

**Unexpected evolutionarily-conserved rapid effects of viral infection on oxytocin receptor and TGF-ß/pSmad3.**

Yutong Liu and Irina Conboy

Univerisity of California, Berkeley, Bioengineering

Berkeley, CA 94720

**Additional files:**

**Additional file 1:**

**Figure S1: Additional comparative qRT PCR data.**

**A.** The *in vitro* studies with mouse primary myoblasts that were transduced with shRNA 1, 2, 3 and their mix.

**B.** Comparative qRT PCR on un-transduced myoblasts vs. satellite sells, vs. control shRNA *in vivo* transduced satellite cells.

N=3, *,**, *** $p<0.05$.

**Additional file 2:**

**Figure S2: Dose curve and timing of OXTR down-regulation by control viruses.**

**A.** Non-target shRNA and empty vector particles down-regulate OXTR in a dose-dependent manner. OXTR downregulation increases with viral dosage with both empty vector particles and non-target shRNA vectors. Myoblasts transduced with non-target shRNAs were collected at 72 hours, and those transduced with empty vector particles at 120 hours. OXTR downregulation is significant even at low viral dosage. n=3 for each cohort. ***$p<0.05$.

**B.** Empty vector particles take longer to decrease OXTR expression compared with non-target shRNAs, but in both cases significant down-regulation is detected by 120 hours post transduction. n=3 for each cohort and each time point. ***$p<0.05$.

**Additional file 3:**

**Figure S3:** High sequence homology between human and mouse Smad3 loci in the area targeted by shRNA is observed and is demonstrated by the alignment.

**Additional file 4:**

**Figure S4: Lentiviral transfection down regulates OXTR as determined by the immunofluorescence.**

Immunofluorescence for OXTR (green) was performed with un-transduced primary human myoblasts versus human myoblasts transduced with control lentiviral shRNA vectors. Hoechst (Blue) labels all nuclei. OXTR specific immunofluorescence was diminished in primary human myoblasts transfected with non-targeted shRNA vectors, as compared to the un-transduced cells.

**Additional file 5:**

**Figure S5: Gel confirmation of qRT PCR results.**

Amplification products from the real-time RT-PCR reactions were run on a gel, and the pixel intensities of each band were quantified by normalizing to *GAPDH*. * $p \leq 0.05$; ** $p \leq 0.01$.

**Supplementary Figures**

**Figure S1A**

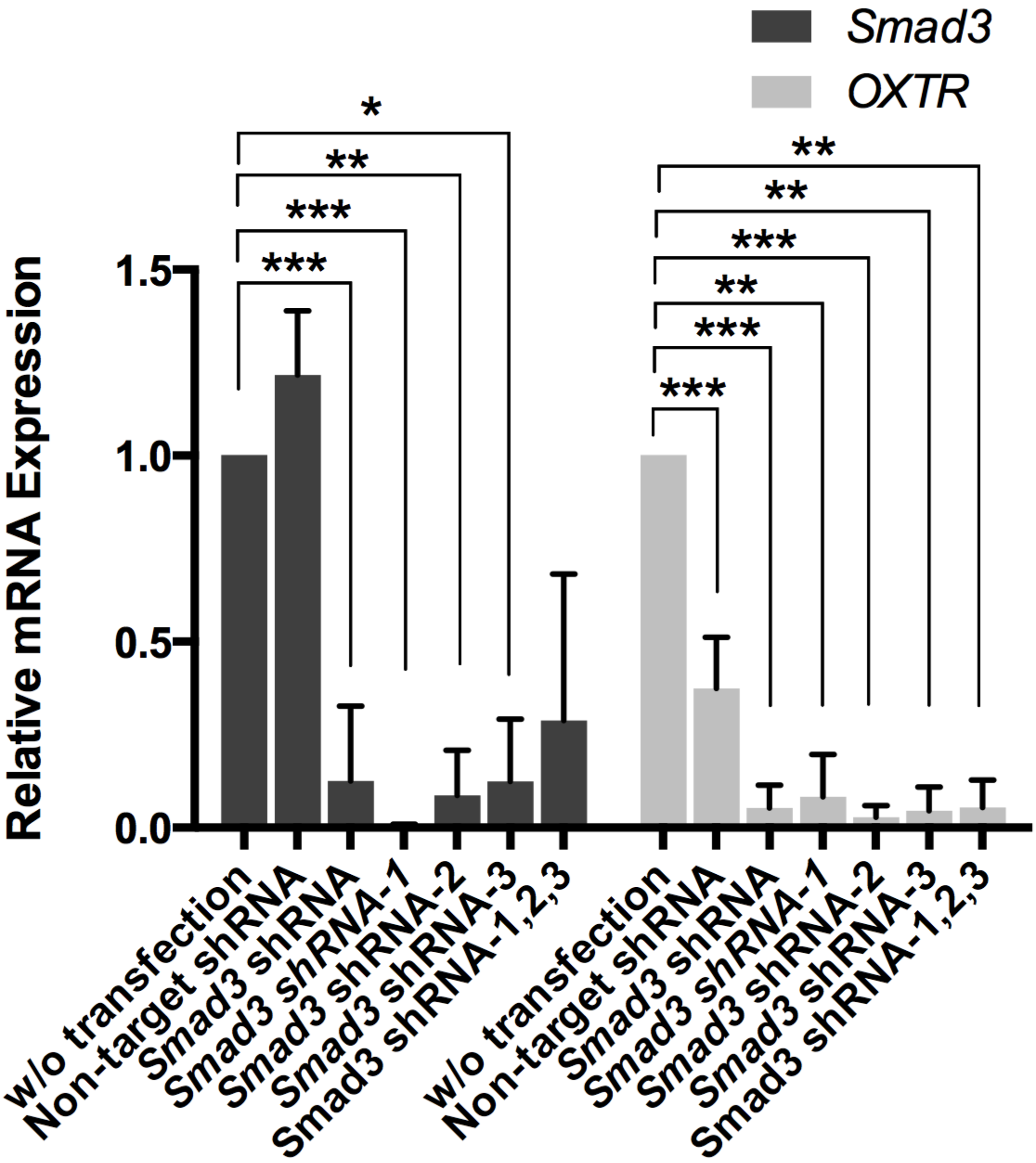

**Figure S1B**

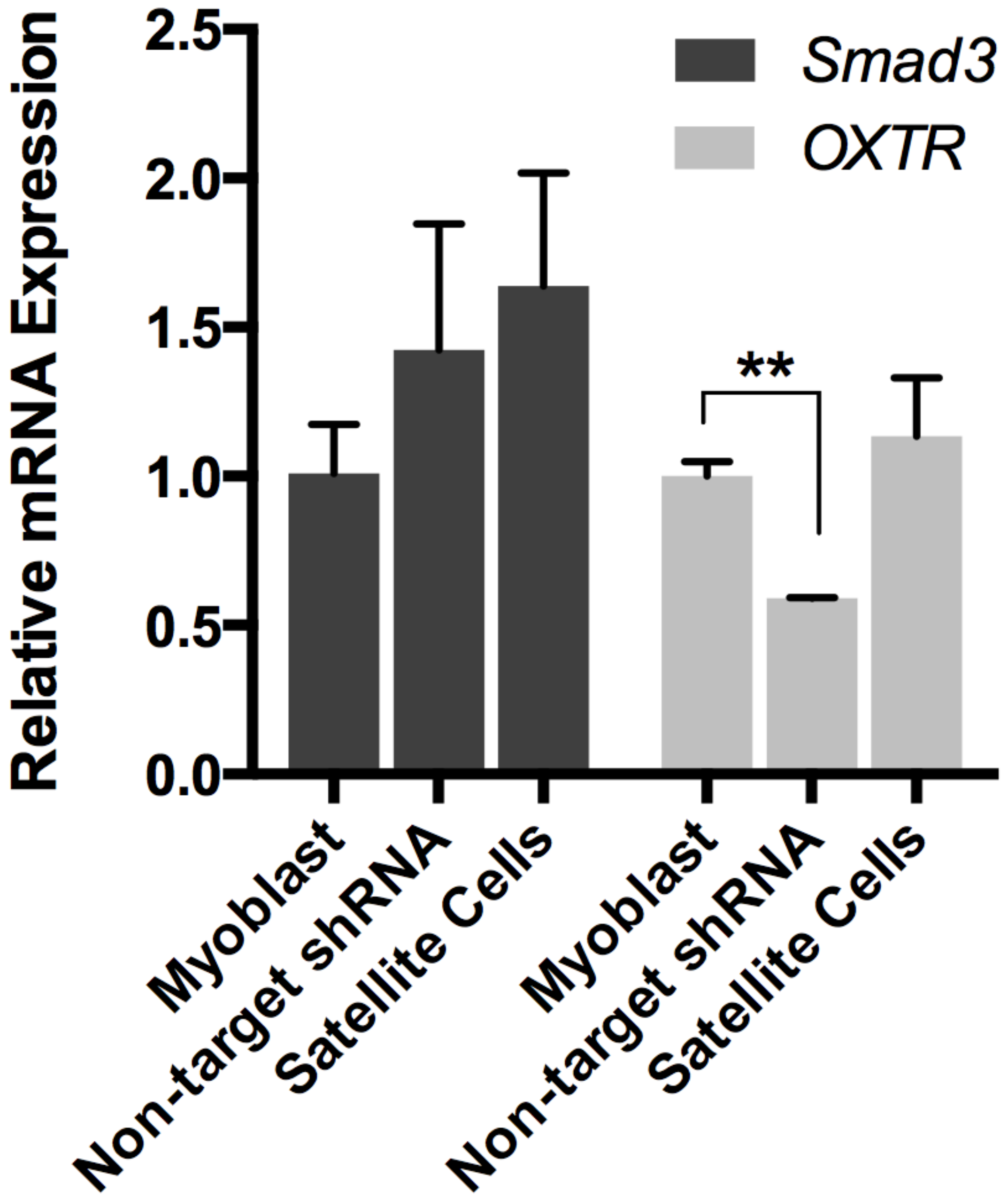

Relative mRNA Expression
2.5
2.0
1.5
1.0
0.5
0.0
Smad3
OXTR
**
Myoblast
Non-target shRNA
Satellite Cells
Myoblast
Non-target shRNA
Satellite Cells

**Figure S2A**

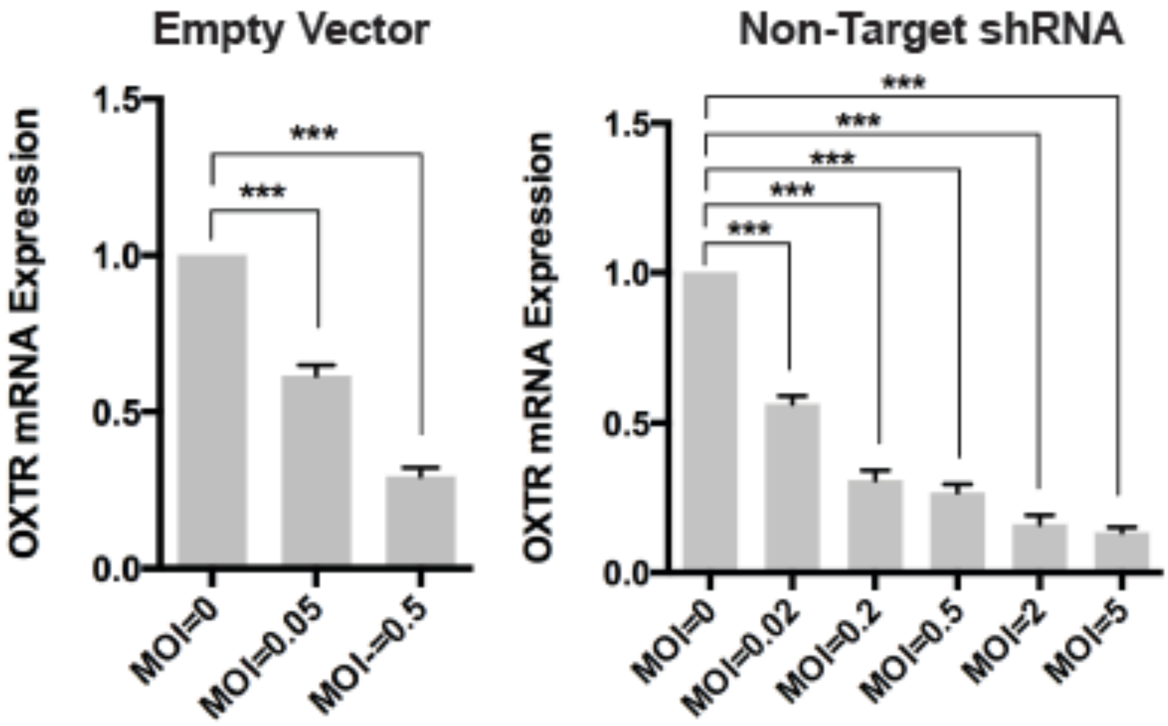

Empty Vector
OXTR mRNA Expression
1.5
1.0
0.5
0.0
***
***
MOI=0
MOI=0.05
MOI-=0.5
Non-Target shRNA
OXTR mRNA Expression
1.5
1.0
0.5
0.0
***
***
***
***
***
MOI=0
MOI=0.02
MOI=0.2
MOI=0.5
MOI=2
MOI=5

**Figure S2B**

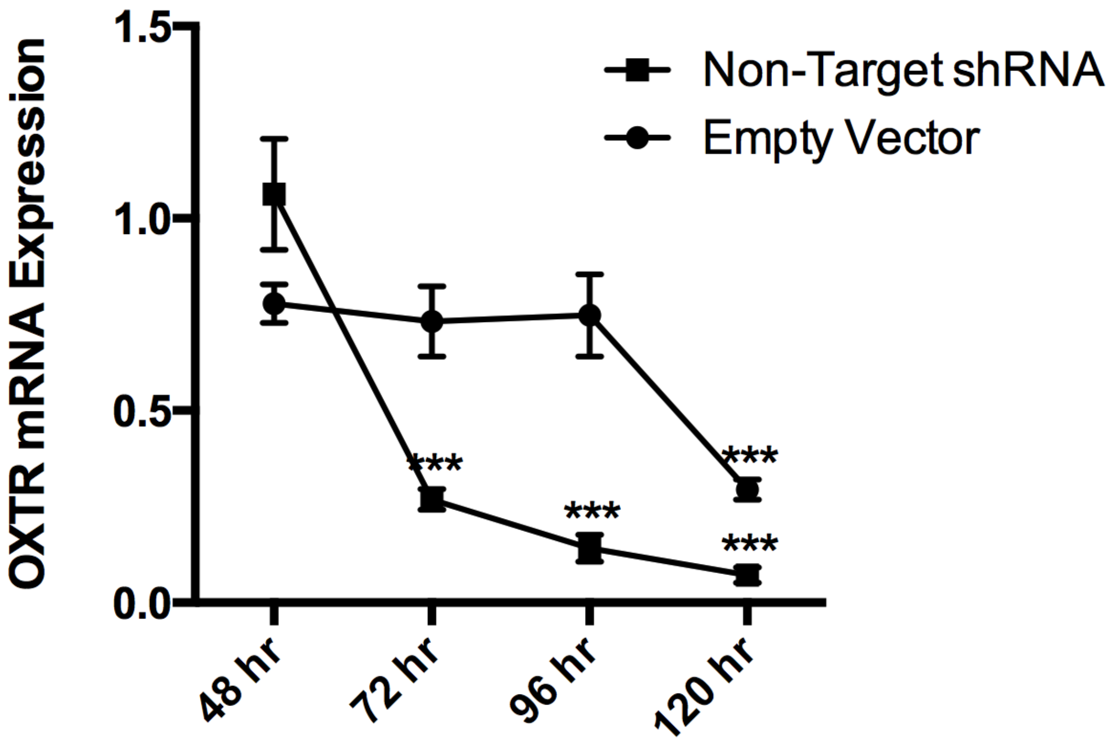

OXTR mRNA Expression
1.5
1.0
0.5
0.0
Non-Target shRNA
Empty Vector
***
***
***
***
48 hr
72 hr
96 hr
120 hr

**Figure S3**

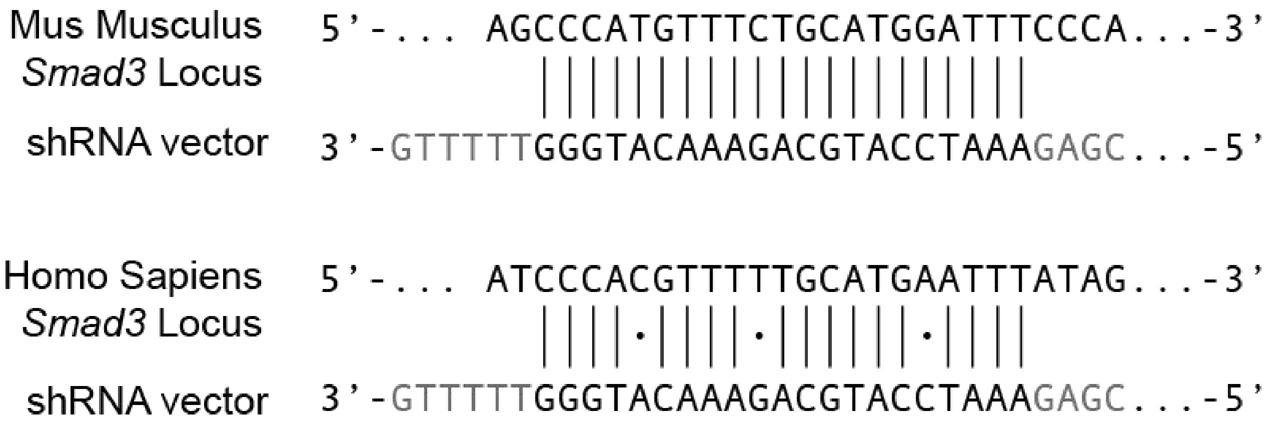
Mus Musculus
*Smad3* Locus
5’-... AGCCCATGTTTCTGCATGGATTTCCCA...-3’
shRNA vector
3’-GTTTTTGGGTACAAAGACGTACCTAAAGAGC...-5’
Homo Sapiens
*Smad3* Locus
5’-... ATCCCACGTTTTTGCATGAATTTATAG...-3’
shRNA vector
3’-GTTTTTGGGTACAAAGACGTACCTAAAGAGC...-5’

**Figure S4**

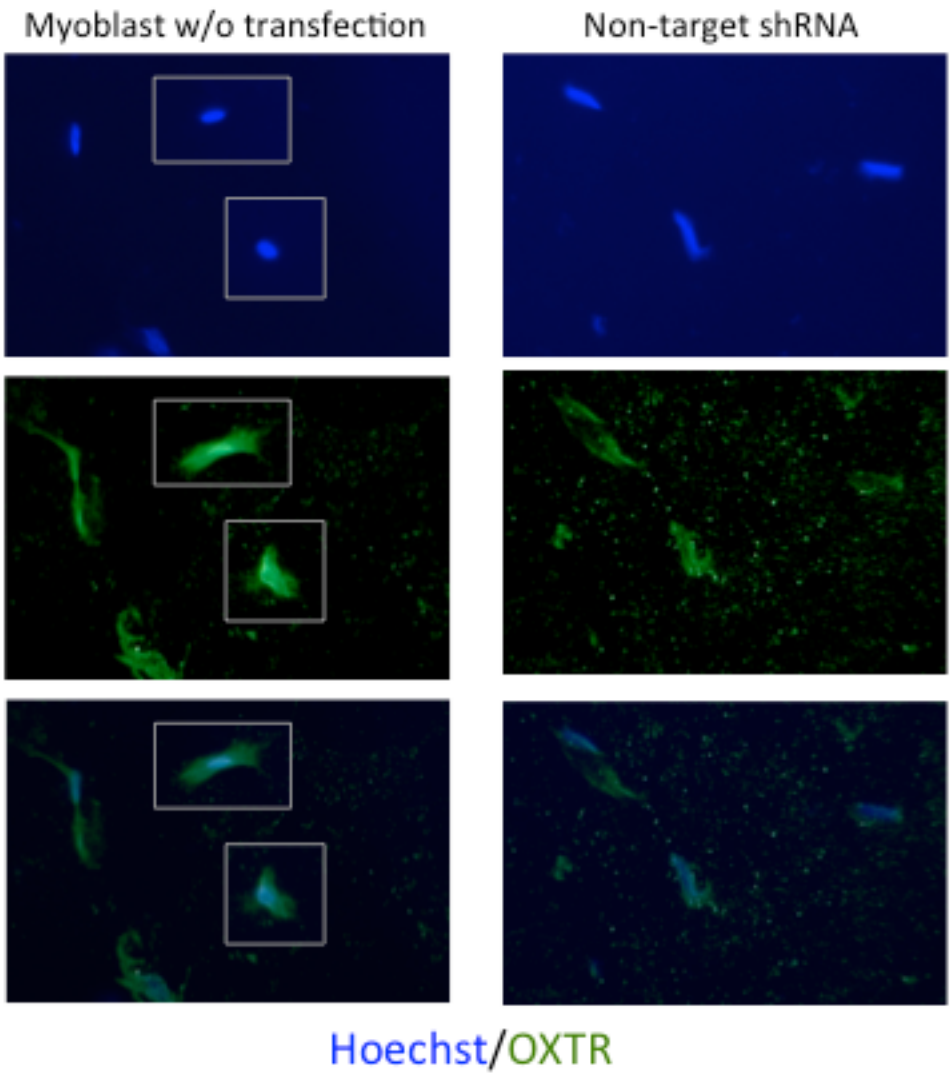

**Figure S5**

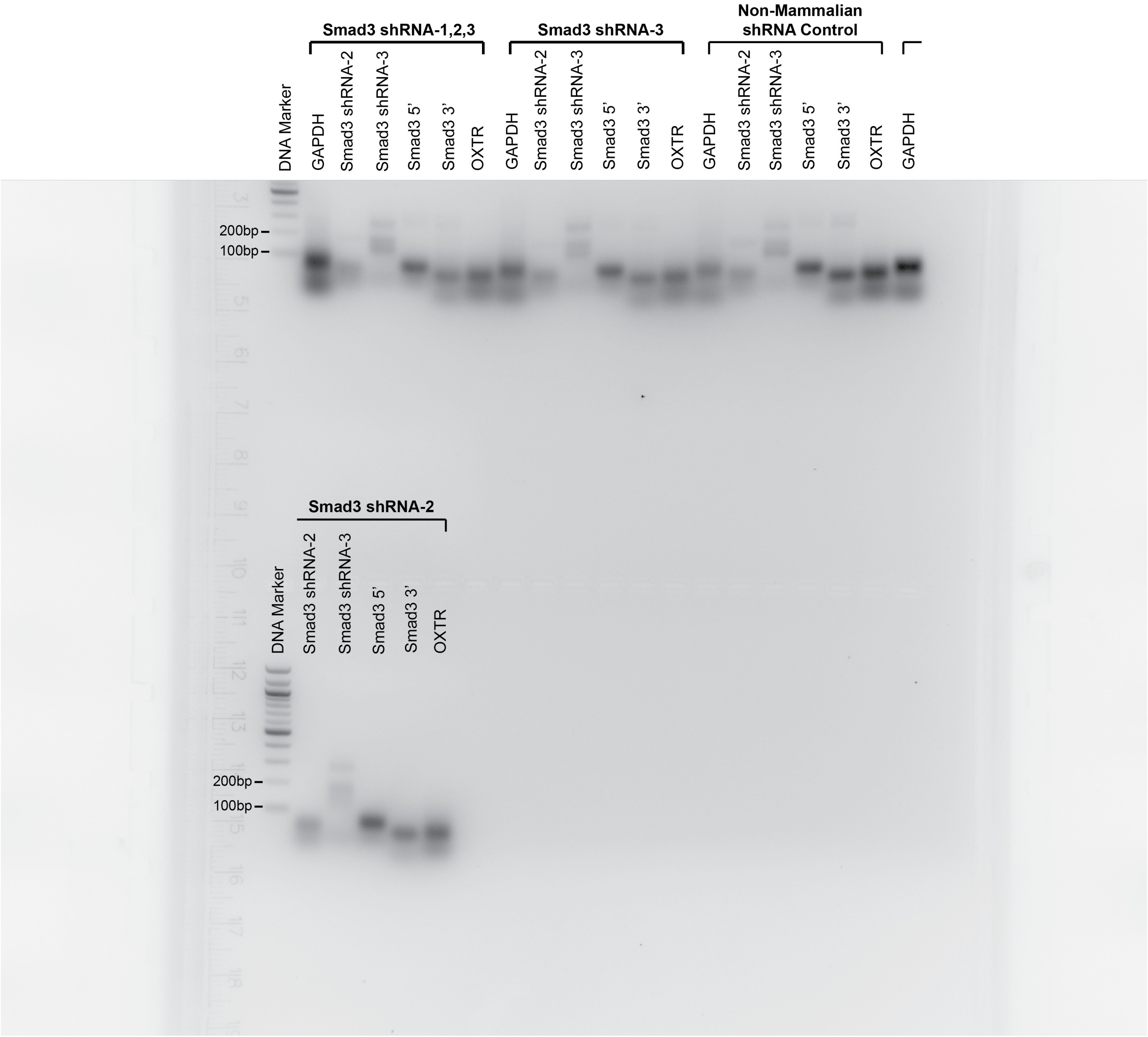
Smad3 shRNA-1,2,3
Smad3 shRNA-3
Non-Mammalian shRNA Control
DNA Marker
GAPDH
Smad3 shRNA-2
Smad3 shRNA-3
Smad3 5'
Smad3 3'
OXTR
200bp
100bp
Smad3 shRNA-2
